# Quantification of Myocardial Contraction Fraction with Three-Dimensional Automated, Machine-Learning-Based Left-Heart-Chamber Metrics: Diagnostic Utility in Hypertrophic Phenotypes and Normal Ejection Fraction

**DOI:** 10.3390/jcm12175525

**Published:** 2023-08-25

**Authors:** Andrea Barbieri, Jacopo F. Imberti, Mario Bartolomei, Niccolò Bonini, Vera Laus, Laura Torlai Triglia, Simona Chiusolo, Marco Stuani, Chiara Mari, Federico Muto, Ilaria Righelli, Luigi Gerra, Mattia Malaguti, Davide A. Mei, Marco Vitolo, Giuseppe Boriani

**Affiliations:** 1 Cardiology Division, Department of Biomedical, Metabolic and Neural Sciences, Policlinico di Modena, University of Modena and Reggio Emilia, 41124 Modena, Italy; 2 Clinical and Experimental Medicine PhD Program, University of Modena and Reggio Emilia, 41124 Modena, Italy

**Keywords:** 3D echocardiography, artificial intelligence, cardiac chamber quantification, machine learning

## Abstract

**Aims:** The differentiation of left ventricular (LV) hypertrophic phenotypes is challenging in patients with normal ejection fraction (EF). The myocardial contraction fraction (MCF) is a simple dimensionless index useful for specifically identifying cardiac amyloidosis (CA) and hypertrophic cardiomyopathy (HCM) when calculated by cardiac magnetic resonance. The purpose of this study was to evaluate the value of MCF measured by three-dimensional automated, machine-learning-based LV chamber metrics (dynamic heart model [DHM]) for the discrimination of different forms of hypertrophic phenotypes. **Methods and Results:** We analyzed the DHM LV metrics of patients with CA (*n* = 10), hypertrophic cardiomyopathy (HCM, *n* = 36), isolated hypertension (IH, *n* = 87), and 54 healthy controls. MCF was calculated by dividing LV stroke volume by LV myocardial volume. Compared with controls (median 61.95%, interquartile range 55.43–67.79%), mean values for MCF were significantly reduced in HCM—48.55% (43.46–54.86% *p* < 0.001)—and CA—40.92% (36.68–46.84% *p* < 0.002)—but not in IH—59.35% (53.22–64.93% *p* < 0.7). MCF showed a weak correlation with EF in the overall cohort (R^2^ = 0.136) and the four study subgroups (healthy adults, R^2^ = 0.039 IH, R^2^ = 0.089; HCM, R^2^ = 0.225; CA, R^2^ = 0.102). ROC analyses showed that MCF could differentiate between healthy adults and HCM (sensitivity 75.9%, specificity 77.8%, AUC 0.814) and between healthy adults and CA (sensitivity 87.0%, specificity 100%, AUC 0.959). The best cut-off values were 55.3% and 52.8%. **Conclusions:** The easily derived quantification of MCF by DHM can refine our echocardiographic discrimination capacity in patients with hypertrophic phenotype and normal EF. It should be added to the diagnostic workup of these patients.

## 1. Introduction

When conventional endocardial measurements of ejection fraction (EF) are used to assess left ventricular (LV) function, the increases in wall thickness or decreases in diameters might mask the existence of a significant impairment in systolic performance [1,2]. Experimental [3,4] and clinical studies using strain analysis [5] have demonstrated that less fiber shortening is required to achieve a comparable EF in a thick-walled compared to a thin-walled LV. Similarly, despite comparable shortening and wall thickness, a smaller LV will have a greater EF than a larger ventricle [1]. Therefore, in clinical practice, it is essential to have access to metrics that, unlike the assessment of EF, can identify meaningful myocardial disease in individuals with a hypertrophic phenotype [6].

One of the most intriguing parameters in this context is the ratio of stroke volume to myocardial volume, which determines the myocardial contraction fraction (MCF). It is a measure of how much the myocardium contracts during systole (as measured by stroke volume [SV]) in comparison to its ability to contract (as measured by myocardial volume) [7,8].

There is ample evidence that the MCF can discriminate between pathological and normal hypertrophy [1,9,10] and identify individuals with heart failure with preserved EF due to amyloidosis from those with increased wall thickness due to other conditions [11,12]. Prognostically, the MCF outperforms the EF when measured by cardiac magnetic resonance in patients with cardiac amyloidosis (CA) or hypertrophic cardiomyopathy (HCM) [9,13]. However, one notable disadvantage of MCF measurement is that it is not a standard component of transthoracic echocardiography since the LV mass quantification using the standard linear diameter measures is frequently inaccurate in hearts with regional heterogeneity [14]. Furthermore, the systematic underestimation of the LV volumes assessment by 2-dimensional echocardiography (2DE) compared to cardiac magnetic resonance further complicates the routine use of MCF [15].

Yet, many of these issues are now solved by automated three-dimensional echocardiography (3DE) [16]. Recently, a further improvement in 3DE technology provided a new algorithm for LV analysis, which is based on the principles of machine learning dynamic heart model ([DHM], Philips Healthcare, Andover, MA, USA) using a training set of over a thousand studies [17], allowing for a feasible, fast, accurate, and reproducible automated quantification of LV mass [18] and volumes [19] in one single output. Therefore, applying machine learning techniques to 3DE has great potential to enhance physician familiarity with the MCF metric in clinical practice. However, despite the high accuracy, a DHM measure of MCF has not been described yet.

To address this, we describe the measure of MCF by DHM and compare it in normal subjects in patients with isolated hypertension, CA, and HCM.

## 2. Materials and Methods

### 2.1. Study Subjects

The study population comprised patients aged ≥18 who underwent standard transthoracic Doppler echocardiography for any indication from 14 September 2020 to 9 November 2021 at Modena University Hospital’s echocardiography laboratory. Criteria for enrollment included age ≥18 years, complete resting 2D and 3D echocardiographic assessment, and EF ≥ 50%. We excluded patients with unsatisfactory images—the margins were not seen well and thus deemed untraceable, patients on dialysis or end-stage liver failure, since large fluid shifts in these patients may cause significant extemporaneous differences in LA and LV measurements, patients with moderate or severe valvular heart disease, pregnant women and those who are in the six months following childbirth, and patients with morbid obesity or leanness (BMI ≥ 30 kg/m^2^, BMI < 18.5 kg/m^2^, respectively).

**Figure 1:**
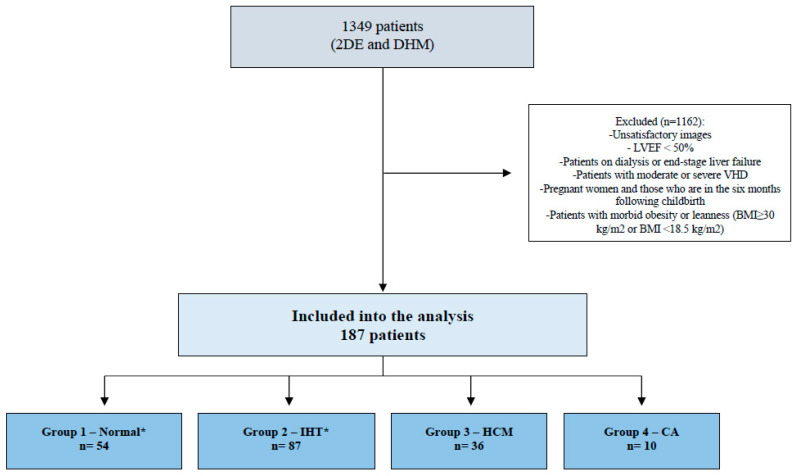
Flowchart depicting the patient selection process. * Absence of diabetes, hypertension, previous or current heart disease, cardiac implantable electronic devices, stroke, chronic obstructive pulmonary disease, venous thromboembolism, cancer, previous or current systemic diseases that may have an impact on the cardiovascular system, drug therapy, or other treatments with cardiovascular effects, normal cardiovascular physical examination, normal ECG at rest) without clinical indication of DHM. 2DE: bidimensional echocardiography; DHM: Dynamic Heart Model; LVEF: left ventricular ejection fraction; VHD: valvular heart disease; BMI: body mass index; IHT: isolated arterial hypertension; HCM: hypertrophic cardiomyopathy; CA: cardiac amyloidosis.

Then, we identify four groups of subjects (Figure 1): (1) healthy adults (absence of diabetes, hypertension, previous or current heart disease, cardiac implantable electronic devices, stroke, COPD, venous thromboembolism, cancer, previous or current systemic diseases that may have an impact on the cardiovascular system, drug therapy or other treatments with cardiovascular effects, normal cardiovascular physical examination, normal ECG at rest) without clinical indication on DHM, (2) isolated hypertension (same requirements as for normal group, but with only a history of systemic arterial hypertension), (3) patients with HCM, diagnosed according to current guidelines [20,21] (4) patients with CA, diagnosed according to the position statement of the European Society of Cardiology Working Group on Myocardial and Pericardial Diseases [22].

Age, sex, height, weight, body surface area (BSA), cardiac rhythm, clinical indications, and history of cardiovascular diseases were recorded at the time of the echocardiography. The study protocol followed the ethical guidelines of the 1975 Declaration of Helsinki and was approved by the local ethic committee (Protocol Code: 234-2021, date of approval: 11 May 2021).

### 2.2. Echocardiographic Data

A complete 2D and 3D transthoracic echocardiographic examination was performed, according to current guidelines [23,24], using a commercial ultrasound system (EPIQ CVx, Philips Healthcare) equipped with an X5-1 transducer. We used a single-beat acquisition mode and multiple cardiac cycles (3–5 beats) in patients with atrial fibrillation. Analysis of DHM methodology was described in detail in our recent publication [25]. Briefly, after setting gain, time-gain compensation, and depth on 2D images, a single-beat acquisition mode from the apical four-chamber view was used to acquire 3D wide-angle datasets. By changing sector width and image depth, the 3D frame rate was optimized. All the acquisitions were performed by operators fully trained in echocardiography with long-standing experience with the 3D technique and trained on echocardiographic datasets focusing on what constitutes adequate automated analysis. The novel vendor software simultaneously detects LV and left atrial (LA) endocardial surfaces using an adaptive analytics algorithm, which uses knowledge-based identification to orient and locate cardiac chambers and patient-specific adaptation of endocardial borders from which LV and LA volumes are derived directly without geometrical assumptions.

Using the automated DHM program, which automatically detects LV endo- and epicardial borders at the end-diastole, 3D-LV mass was analyzed, enabling direct LV mass thickness. Although it is possible to correct the LV and LA endocardial–epicardial borders at the end-diastole and and-systole, manual border adjustments were performed when deemed indicated by the operator. We considered the following DHM measures: LV end-diastolic volume indexed to BSA (EDVi), LV end-systolic volume indexed to BSA (ESVi), EF, SV, LA maximum volume indexed to BSA (LAVi max), LA minimum volume indexed to BSA (LAVi min), LA ejection fraction (LAEF), automatically calculated as LA maximum volume-LA minimum volume/LA maximum volume), LV mass, LV mass indexed to BSA, LV mass: LV end-diastolic volume ratio (LVM/LVEDV). We considered the MCF as an additional measure of the LV function as LV stroke volume/LV myocardial volume × 100. The myocardial volume was assessed by directly measuring the LV mass between the epicardium and endocardium by DHM and calculated by dividing the LV mass by 1.05 (the density of myocardial tissue). When correctly performed, the calculation of LV myocardial volume is the same whether performed during systole or diastole because myocardial tissue, being not compressible, does not change during contraction [26]. All 3D echocardiography images were analyzed online using the larger default boundary detection sliders (end-diastolic position = 60/60; end-systolic position = 30/30). This setting defines diastolic and systolic contour positions within the myocardial wall ranging from 0 to 100, 0 being the most inner endocardial contour toward the LV cavity and 100 being the most outer endocardial contour towards the myocardial wall. To our knowledge, there are no specific recommendations about this feature, and the decision is left to the single image laboratory. We deliberately decided to apply these fixed threshold borders settings because they were the ones used in previous validation studies, and they are closer to the settings of CMR [27]. All measurements were entered into an electronic database during the echocardiographic examination. No modification from the original database was applied, and no 3D measurement was made offline. Hence, the study consisted of a retrospective analysis of data prospectively included in the electronic echocardiographic database.

## 3. Statistical Analysis

Data are shown as counts and percentages or median and interquartile range (IQR). Categorical variables were compared with the Chi-square test (or Fisher’s exact test if appropriate). For continuous variables, a comparison was made with the Mann–Whitney U test. Linear regression analysis was used to compare MCF and LVEF. The quality standards for correlations were defined as: very good: 0.8 < R^2^ < 1.0; good: 0.6 < R^2^ < 0.8; moderate: 0.4 < R^2^ < 0.6; or poor: R^2^ < 0.4. The discriminatory performance of the MCF for each study subgroup was explored using receiver-operating characteristic (ROC) curves and the area under the curve (AUC) analysis; the Youden index was used to determine the best cut-off values. A *p*-value < 0.05 was considered to be statistically significant in all the analyses. Analyses were performed using SPSS^®^ version 26 (IBM Corp, Armonk, NY, USA).

## 4. Results

The original dataset consisted of 1349 consecutive patients. Among them, we identified 54 healthy adults, 87 patients affected by isolated hypertension, 36 by HCM, and 10 by CA. Median age was 49 (37–69) years, 61 (52–75) years, 63 (56–69) years, and 81 (79–82) years, respectively. Females accounted for 51.9% of healthy adults, 55.2% of isolated hypertension, 32.4% of HCM, and 10% of CA. Of note, 10.8% of HCM patients and 50% of CA patients had a history of atrial fibrillation. Most healthy adults (90.7%) and isolated hypertension (87.4%) showed an MCF ≥ 50%, while this finding was observed only in 41.7% of HCM patients and 10% of CA patients. Patients with MCF ≥ 50% were younger as compared with patients with MCF < 50% (57 (46–70) vs. 72 (59–81) years, *p* < 0.001), they were more frequently female (53.9% vs. 26.1%; *p* < 0.001) and less frequently had a history of coronary artery disease (0.7% vs. 8.7%, *p* = 0.014). A detailed description of the patient’s demographic and clinical characteristics according to their baseline MCF value is shown in Table 1.

**Figure 2:**
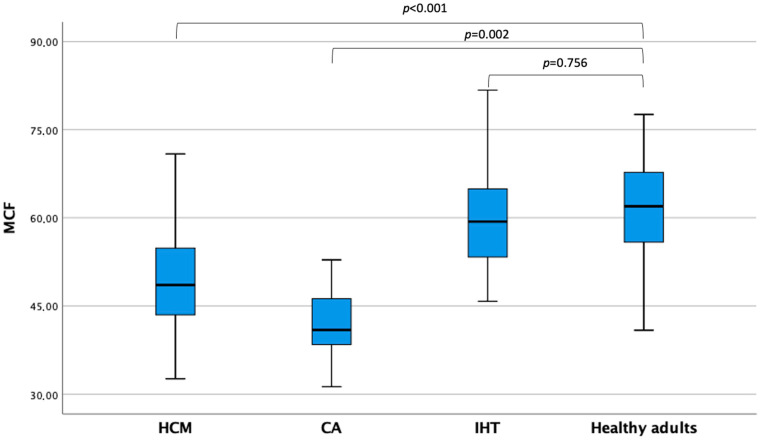
Myocardial contraction fraction values across patients’ subgroups CA, cardiac amyloidosis; HCM, hypertrophic cardiomyopathy; IHT, isolated hypertension; MCF, myocardial contraction fraction.

Echocardiographic parameters are shown in Table 2. Interestingly, LVEDVi and LVESVi were not different between patients with MCF ≥ 50% and <50% (72.3 (62.9–81.8) vs. 68.2 (59.7–82.1) mL/m^2^; *p* = 0.469 and 28.6 (23.0–35.8) vs. 32.0 (24.6–38.7) mL/m^2^; *p* = 0.154, respectively). On the other hand, patients with MCF ≥ 50% showed a significantly lower LAVi max (34.4 (28.4–42.7) vs. 54.0 (41.3–65.3) mL/m^2^; *p* < 0.001) and LAVi min (12.7 (9.4–17.1) vs. 30.3 (17.7–40.2) mL/m^2^; *p* < 0.001) and a higher LAEF (62.0% (56.0–68.0) vs. 45% (29.0–59.0); *p* < 0.001). The median MCF in the overall cohort was 57.34 (50.1–64.9). Healthy adults showed higher MCF values than HCM (*p* < 0.001) and CA patients (*p* = 0.002), while they did not differ as compared with isolated hypertension (*p* = 0.756) (Figure 2).

**Figure 3:**
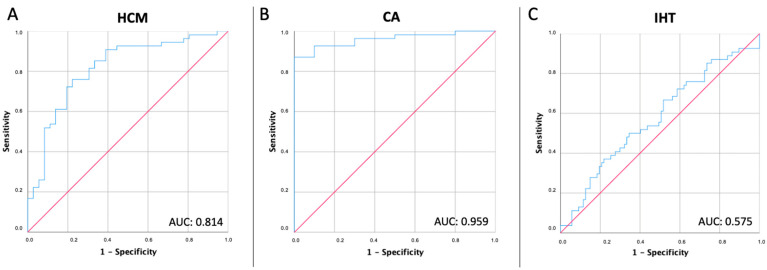
Performance of myocardial contraction fraction for detecting hypertrophic phenotypes. (**Panel A**) Receiver-operating characteristics (ROC) curve showing the performance of myocardial contraction fraction for detecting hypertrophic cardiomyopathy. (**Panel B**) ROC curve showing the performance of myocardial contraction fraction for detecting Cardiac amyloidosis. (**Panel C**) ROC curve showing the performance of myocardial contraction fraction for detecting isolated hypertension. AUC, area under the curve; CA, cardiac amyloidosis; HCM, hypertrophic cardiomyopathy; IHT, isolated hypertension.

MCF showed a weak level of correlation with LVEF in the overall cohort (R^2^ = 0.136) and the four study subgroups as well (healthy adults, R^2^ = 0.039; isolated hypertension, R^2^= 0.089; HCM, R^2^ = 0.225; CA, R^2^ = 0.102). ROC analyses showed that MCF had a high capability to differentiate between healthy adults and HCM patients (sensitivity 75.9%, specificity 77.8%, AUC 0.814) and between healthy adults and CA patients (sensitivity 87.0%, specificity 100%, AUC 0.959), but not between healthy adults and isolated hypertension (sensitivity 50.0%, specificity 65.5%, AUC 0.575). The best cut-off values were 55.37, 52.86, and 62.24, respectively (Figure 3).

## 5. Discussion

**Figure 4:**
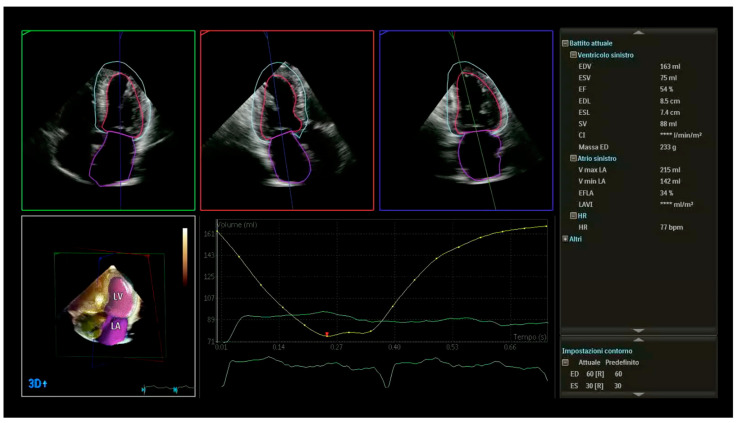
Example of 3D measurements (LV volumes and LV mass) obtained from automated DHM in a patient with cardiac amyloidosis and normal EF (54%). This advanced echocardiography allows for accurate, automated measurements of chamber volumes and function providing larger, more accurate LV volumes with good agreement with cardiac magnetic resonance when analyzed using the default settings of the boundary detection sliders (end-diastolic default position = 60/60; end-systolic default position = 30/30). LV myocardial mass (233 g) divided by the mean density of myocardium of 1.05 g/mL presents LV myocardial volume (221 mL). The ratio of SV (88 mL) to myocardial volume calculated the Myocardial Contraction Fraction (39%), significantly lower than the EF. LV: left ventricular; DHM: Dynamic Heart Model, Philips Healthcare, Andover, MA, USA; SV: stroke volume; EF: ejection fraction. DHM automatically measures chamber volumes throughout the cardiac cycle, resulting in LV time curve (yellow).

The MCF is a dimensionless index with a clear, simple definition supported by proof of a predictive value when calculated by cardiac magnetic resonance [9,12,28,29,30]. The primary finding of this study was that in patients with normal EF, the MCF by DHM clearly distinguishes the groups of normal subjects and patients with isolated hypertension from the groups of patients with HCM and CA. Despite similar degrees of hypertrophy between isolated hypertensive and HCM groups, we found that the MCF was significantly different. The MCF decreased in patients with HCM and even more in patients with CA, reflecting a relatively greater decrease in SV than myocardial volume (Figure 4). Of note, in our cohort, patients with abnormal MCF (<50%) demonstrated a higher hemodynamic burden (increased LAVi max, LAVi min, decreased LAEF) compared with patients with normal MCF, which strengthens our evidence even more. Thus, the MCF by DHM appears as an additional appealing echocardiographic parameter in patients with hypertrophic phenotypes.

The differentiation of pathologic hypertrophy is challenging in patients with mild forms of hypertrophy and normal EF. We showed that the MCF was not significantly different among normal subjects and isolated hypertensive patients. The lack of statistically significant difference may be due to the small sample size in combination with nonadvanced hypertensive heart disease and the lack of the superimposition of clinical heart failure in the group with isolated hypertension. A previous study showed that the MCF by manual 3DE could differentiate subjects with hypertensive hypertrophy and heart failure from endurance athletes with hypertrophy [7].

Echocardiography is a first-line screening tool for patients with LV hypertrophy and normal EF. In both HCM and CA, both the SV and LVEDV are reduced. Therefore, the calculated ratio of SV to LVEDV (i.e., the EF) typically falls into the normal or near-normal range even though myocardial shortening and systolic function are significantly hampered [31]. As a result, cardiomyocyte function abnormalities should be detected using measurements of myocardial shortening (i.e., MCF and strain) [9,10,11,12]. Pagourelias et al. showed that the deformation parameters differentiate better CA from other hypertrophic substrates than MCF calculated by 2DE [11]. However, it is worth noting that the MCF requires precise SV and myocardial volume measurement. Therefore, its use in clinical practice should be restricted to 3DE since estimates of these parameters obtained by 2DE techniques are neither sufficiently accurate nor reproducible to be comparable to 3DE results [32,33]. Conversely, DHM provides a multiparametric output quickly at the same time (i.e., volume and function), in conjunction with the proven accuracy and reproducibility of LV mass and volumes [18,19], allowing for the broadly reliable MCF application in clinical practice.

## 6. Limitations

Our study was retrospective in design, and the sub-analysis of the patients with HCM and CA was restricted to a small group. However, all our groups were strictly defined, and the number of patients included is similar compared with other studies investigating LV hypertrophy differential diagnosis.

The healthy sedentary subjects and those involved in competitive sports were not differentiated. Indeed, the MCF is increased in subjects with physiologic hypertrophy, reflecting a relatively greater increase in SV than myocardial volume [7].

We do not account for LV global longitudinal strain because it was not feasible in a non-negligible percentage of the sample analyzed. However, conceptually, the MCF is comparable to myocardial strain because it is a generalized measure of myocardial shortening [5]. Yet, the MCF combines information on shortening from all domains (longitudinal, radial, and circumferential) into a single value [8], whereas strain quantifies shortening in a particular section of the heart [31]. Nevertheless, more data are required to assess their reciprocal diagnostic benefit.

A more appropriately powered study examining associations would allow for a more granular disease-specific sub-analysis highlighting eventual variations of the MCF concerning HCM phenotypes, different stages of CA, and hypertensive heart disease. This issue may contribute to future studies on disease stages and risk stratification. Indeed, the probability of MCF becoming abnormal gradually increases across the spectrum of CA deposition [2].

The MCF by DHM was not performed on top of conventional 2DE evaluation for comparative analysis [34,35,36,37]. However, since the DHM has been available, our echo lab has stopped performing 2DE volumetric analysis regularly. Moreover, our group recently showed that the LV mass assessment by DHM showed systematic differences and wide limits of agreements compared with the standard 2DE quantification [38].

We were unable to gather more detailed information on the history and severity of hypertension since this was a retrospective analysis of a group that had transthoracic echocardiography.

Finally, although easy to calculate, MCF by DHM is still a derived parameter. Therefore, postprocessing algorithms should hopefully be developed quickly to estimate MCF within an even shorter time delay regarding transferability to the clinical practice.

## 7. Conclusions

The present data suggested that the quantification of the MCF by DHM can refine our echocardiographic discrimination capacity in patients with hypertrophic phenotype and normal EF. The following steps will require assessing its utility in other groups and different clinical scenarios for more general application in clinical practice.

## Figures and Tables

**Table 1:** Baseline characteristics of the overall cohort according to myocardial contraction fraction values.

	MCF ≥ 50% (*n* = 141)	MCF < 50% (*n* = 46)	Total (*n* = 187)	*p*-Value
Age, years	57.00 (46.0–70.0)	71.50 (59.0–81.0)	61.00 (48.3–72.8)	<0.001
Female	76 (53.9)	12 (26.1)	88 (47.1)	<0.001
Heart rate, bpm	69.00 (62.0–77.0)	67.50 (60.8–81.5)	69.00 (62.0–78.0)	0.791
Hypertension	78 (55.3)	21 (45.7)	99 (52.9)	0.308
Diabetes	0.0 (0.0)	2 (4.3)	2 (1.1)	0.060
CAD	1 (0.7)	4 (8.7)	5 (2.7)	0.014
ACS	1 (0.7)	2 (4.3)	3 (1.6)	0.150
Stroke/TIA	1 (0.7)	1 (2.2)	2 (1.1)	0.432
Heart failure	0 (0.0)	0 (0.0)	0 (0.0)	NA
Atrial fibrillation	1 (0.7)	8 (17.4)	9 (4.8)	<0.001
CIED	2 (1.4)	5 (10.9)	7 (3.7)	0.004
Pulmonary embolism	0 (0.0)	1 (2.2)	1 (0.5)	0.246
Healthy adults	49 (90.7)	5 (9.3)	54 (100)	<0.001
Isolated hypertension	76 (87.4)	11 (12.6)	87 (100)	
Hypertrophic cardiomyopathy	15/36 (41.7)	21/36 (58.3)	36736 (100)	
Cardiac amyloidosis	1 (10.0)	9 (90.0)	10 (100)	

Values are median (IQR) and *n* (%). ACS, acute coronary syndrome; Bpm, beats per minute; CAD, coronary artery disease; CIED, cardiac implantable electronic devices; NA, not applicable; TIA, transient ischemic attack.

**Table 2:** Baseline characteristics of the overall cohort according to myocardial contraction fraction values.

	MCF ≥ 50% (*n* = 141)	MCF < 50% (*n* = 46)	Total (*n* = 187)	*p*-Value
**EDD, mm**	48.00 (44.00–51.00)	44.00 (40.75–49.25)	47.00 (44.00–51.00)	0.006
**LV Mass-i (2D), g**	73.96 (62.60–90.81)	114.49 (75.18–141.48)	76.65 (63.17–99.92)	<0.001
**LV Mass-i, g/m^2^**	73.81 (63.63–82.93)	93.25 (84.11–109.14)	77.37 (67.05–89.47)	<0.001
**LV EDVi (2D), mL/m^2^**	53.99 (53.18–58.62)	54.79 (50.27–60.47)	53.99 (53.31–58.05)	0.967
**LV EDVi, mL/m^2^**	72.27 (62.88–81.78)	68.20 (59.72–82.12)	71.82 (62.32–81.66)	0.469
**LV ESVi, mL/m^2^**	28.60 (22.96–35.81)	31.97 (24.60–38.70)	28.71 (23.49–36.04)	0.154
**SVi, g/m^2^**	42.71 (37.46–48.87)	39.69 (33.94–44.48)	42.06 (36.66–48.32)	0.006
**LVEF, %**	60.00 (56.00–63.50)	56.00 (51.75–60.25)	58.50 (55.00–63.00)	<0.001
**MCF**	60.43 (55.28–66.44)	44.99 (40.11–47.94)	57.34 (50.06–64.88)	<0.001
**LAVi Max, mL/m^2^**	34.37 (28.35–42.66)	54.01 (41.25–65.25)	37.13 (29.52–48.59)	<0.001
**LAVi Min, mL/m^2^**	12.72 (9.43–17.06)	30.25 (17.74–40.24)	14.33 (10.10–23.34)	<0.001
**LAEF, %**	62.00 (56.00–68.00)	45.00 (29.00–59.00)	60.00 (51.00–66.00)	<0.001

Values are median (IQR) and *n* (%). Measurements, except diameters, were taken using the 3D-DHM if not specified otherwise. EDD, end-diastolic diameter; EDVi, end-diastolic volume index; ESVi, end-systolic volume index; LAEF, left atrial ejection fraction; LAVi Max, left atrial maximum volume index; LAVi Min, left atrial minimum volume index; LV, left ventricular; LVEF, left ventricular ejection fraction; MCF, myocardial contraction fraction; SVi, stroke volume index.

## Data Availability

The data presented in this study are available on reasonable request from the corresponding author.

## References

[1] Stokke T.M., Hasselberg N.E., Smedsrud M.K., Sarvari S.I., Haugaa K.H., Smiseth O.A., Edvardsen T., Remme E.W. (2017). Geometry as a Confounder When Assessing Ventricular Systolic Function: Comparison Between Ejection Fraction and Strain. J. Am. Coll. Cardiol..

[2] Knight D.S., Zumbo G., Barcella W., Steeden J.A., Muthurangu V., Martinez-Naharro A., Treibel T.A., Abdel-Gadir A., Bulluck H., Kotecha T. (2019). Cardiac Structural and Functional Consequences of Amyloid Deposition by Cardiac Magnetic Resonance and Echocardiography and Their Prognostic Roles. JACC Cardiovasc. Imaging.

[3] Capasso J.M., Strobeck J.E., Sonnenblick E.H. (1981). Myocardial mechanical alterations during gradual onset long-term hypertension in rats. Am. J. Physiol..

[4] Mann D.L., Urabe Y., Kent R.L., Vinciguerra S., Cooper G. (1991). Cellular versus myocardial basis for the contractile dysfunction of hypertrophied myocardium. Circ. Res..

[5] Maciver D.H. (2012). The relative impact of circumferential and longitudinal shortening on left ventricular ejection fraction and stroke volume. Exp. Clin. Cardiol..

[6] Borlaug B.A., Sharma K., Shah S.J., Ho J.E. (2023). Heart Failure with Preserved Ejection Fraction: JACC Scientific Statement. J. Am. Coll. Cardiol..

[7] King D.L., El-Khoury Coffin L., Maurer M.S. (2002). Myocardial contraction fraction: A volumetric index of myocardial shortening by freehand three-dimensional echocardiography. J. Am. Coll. Cardiol..

[8] Matthews S.D., Rubin J., Cohen L.P., Maurer M.S. (2018). Myocardial Contraction Fraction: A Volumetric Measure of Myocardial Shortening Analogous to Strain. J. Am. Coll. Cardiol..

[9] Arenja N., Fritz T., Andre F., Riffel J.H., aus dem Siepen F., Ochs M., Paffhausen J., Hegenbart U., Schönland S., Müller-Hennessen M. (2017). Myocardial contraction fraction derived from cardiovascular magnetic resonance cine images-reference values and performance in patients with heart failure and left ventricular hypertrophy. Eur. Heart J. Cardiovasc. Imaging.

[10] Shimada Y.J., Hoeger C.W., Latif F., Takayama H., Ginns J., Maurer M.S. (2019). Myocardial Contraction Fraction Predicts Cardiovascular Events in Patients with Hypertrophic Cardiomyopathy and Normal Ejection Fraction. J. Card. Fail..

[11] Pagourelias E.D., Mirea O., Duchenne J., Van Cleemput J., Delforge M., Bogaert J., Kuznetsova T., Voigt J.-U. (2017). Echo Parameters for Differential Diagnosis in Cardiac Amyloidosis: A Head-to-Head Comparison of Deformation and Nondeformation Parameters. Circ. Cardiovasc. Imaging.

[12] Rubin J., Steidley D.E., Carlsson M., Ong M.L., Maurer M.S. (2018). Myocardial Contraction Fraction by M-Mode Echocardiography Is Superior to Ejection Fraction in Predicting Mortality in Transthyretin Amyloidosis. J. Card. Fail..

[13] Arenja N., Andre F., Riffel J.H., aus dem Siepen F., Hegenbart U., Schönland S., Kristen A.V., Katus H.A., Buss S.J. (2019). Prognostic value of novel imaging parameters derived from standard cardiovascular magnetic resonance in high risk patients with systemic light chain amyloidosis. J. Cardiovasc. Magn. Reson..

[14] Armstrong A.C., Gidding S., Gjesdal O., Wu C., Bluemke D.A., Lima J.A. (2012). LV mass assessed by echocardiography and CMR, cardiovascular outcomes, and medical practice. JACC Cardiovasc. Imaging.

[15] Dorosz J.L., Lezotte D.C., Weitzenkamp D.A., Allen L.A., Salcedo E.E. (2012). Performance of 3-dimensional echocardiography in measuring left ventricular volumes and ejection fraction: A systematic review and meta-analysis. J. Am. Coll. Cardiol..

[16] Nolan M.T., Thavendiranathan P. (2019). Automated Quantification in Echocardiography. JACC Cardiovasc. Imaging.

[17] Tsang W., Salgo I.S., Medvedofsky D., Takeuchi M., Prater D., Weinert L., Yamat M., Mor-Avi V., Patel A.R., Lang R.M. (2016). Transthoracic 3D Echocardiographic Left Heart Chamber Quantification Using an Automated Adaptive Analytics Algorithm. JACC Cardiovasc. Imaging.

[18] Volpato V., Mor-Avi V., Narang A., Prater D., Gonçalves A., Tamborini G., Fusini L., Pepi M., Patel A.R., Lang R.M. (2019). Automated, machine learning-based, 3D echocardiographic quantification of left ventricular mass. Echocardiography.

[19] Wu V.C., Kitano T., Chu P.H., Takeuchi M. (2023). Left ventricular volume and ejection fraction measurements by fully automated 3D echocardiography left chamber quantification software versus CMR: A systematic review and meta-analysis. J. Cardiol..

[20] Ommen S.R., Mital S., Burke M.A., Day S.M., Deswal A., Elliott P., Evanovich L.L., Hung J., Joglar J.A., Kantor P. (2020). 2020 AHA/ACC Guideline for the Diagnosis and Treatment of Patients with Hypertrophic Cardiomyopathy: Executive Summary: A Report of the American College of Cardiology/American Heart Association Joint Committee on Clinical Practice Guidelines. Circulation.

[21] Elliott P.M., Anastasakis A., Borger M.A., Borggrefe M., Cecchi F., Charron P., Hagege A.A., Lafont A., Limongelli G., Mahrholdt H. (2014). 2014 ESC Guidelines on diagnosis and management of hypertrophic cardiomyopathy: The Task Force for the Diagnosis and Management of Hypertrophic Cardiomyopathy of the European Society of Cardiology (ESC). Eur. Heart J..

[22] Garcia-Pavia P., Rapezzi C., Adler Y., Arad M., Basso C., Brucato A., Burazor I., Caforio A.L.P., Damy T., Eriksson U. (2021). Diagnosis and treatment of cardiac amyloidosis. A position statement of the European Society of Cardiology Working Group on Myocardial and Pericardial Diseases. Eur. J. Heart Fail..

[23] Lang R.M., Badano L.P., Mor-Avi V., Afilalo J., Armstrong A., Ernande L., Flachskampf F.A., Foster E., Goldstein S.A., Kuznetsova T. (2015). Recommendations for cardiac chamber quantification by echocardiography in adults: An update from the American Society of Echocardiography and the European Association of Cardiovascular Imaging. J. Am. Soc. Echocardiogr..

[24] Mitchell C., Rahko P.S., Blauwet L.A., Canaday B., Finstuen J.A., Foster M.C., Horton K., Ogunyankin K.O., Palma R.A., Velazquez E.J. (2019). Guidelines for Performing a Comprehensive Transthoracic Echocardiographic Examination in Adults: Recommendations from the American Society of Echocardiography. J. Am. Soc. Echocardiogr..

[25] Barbieri A., Albini A., Chiusolo S., Forzati N., Laus V., Maisano A., Muto F., Passiatore M., Stuani M., Torlai Triglia L. (2022). Three-Dimensional Automated, Machine-Learning-Based Left Heart Chamber Metrics: Associations with Prevalent Vascular Risk Factors and Cardiovascular Diseases. J. Clin. Med..

[26] King D.L., Coffin L.E.-K., Maurer M.S. (2002). Noncompressibility of myocardium during systole with freehand three-dimensional echocardiography. J. Am. Soc. Echocardiogr..

[27] Italiano G., Tamborini G., Fusini L., Mantegazza V., Doldi M., Celeste F., Gripari P., Muratori M., Lang R.M., Pepi M. (2021). Feasibility and Accuracy of the Automated Software for Dynamic Quantification of Left Ventricular and Atrial Volumes and Function in a Large Unselected Population. J Clin. Med..

[28] Chuang M.L., Gona P., Salton C.J., Yeon S.B., Kissinger K.V., Blease S.J., Levy D., O’Donnell C.J., Manning W.J. (2012). Usefulness of the left ventricular myocardial contraction fraction in healthy men and women to predict cardiovascular morbidity and mortality. Am. J. Cardiol..

[29] Abdalla M., Akwo E.A., Bluemke D.A., Lima J.A.C., Shimbo D., Maurer M.S., Bertoni A.G. (2019). Association between reduced myocardial contraction fraction and cardiovascular disease outcomes: The Multi-Ethnic Study of Atherosclerosis. Int. J. Cardiol..

[30] Hou X., Xiong X., Li X., Bi J., Xu G., Wang Y., Jiang S. (2022). Predictive value of cardiac magnetic resonance mechanical parameters for myocardial fibrosis in hypertrophic cardiomyopathy with preserved left ventricular ejection fraction. Front. Cardiovasc. Med..

[31] Maurer M.S., Packer M. (2020). How Should Physicians Assess Myocardial Contraction?: Redefining Heart Failure With a Preserved Ejection Fraction. JACC Cardiovasc. Imaging.

[32] Patel H.N., Miyoshi T., Addetia K., Henry M.P., Citro R., Daimon M., Gutierrez Fajardo P., Kasliwal R.R., Kirkpatrick J.N., Monaghan M.J. (2021). Normal Values of Cardiac Output and Stroke Volume According to Measurement Technique, Age, Sex, and Ethnicity: Results of the World Alliance of Societies of Echocardiography Study. J. Am. Soc. Echocardiogr..

[33] Addetia K., Miyoshi T., Amuthan V., Citro R., Daimon M., Gutierrez Fajardo P., Kasliwal R.R., Kirkpatrick J.N., Monaghan M.J., Muraru D. (2022). Normal Values of Left Ventricular Size and Function on Three-Dimensional Echocardiography: Results of the World Alliance Societies of Echocardiography Study. J. Am. Soc. Echocardiogr..

[34] Tendler A., Helmke S., Teruya S., Alvarez J., Maurer M.S. (2015). The myocardial contraction fraction is superior to ejection fraction in predicting survival in patients with AL cardiac amyloidosis. Amyloid.

[35] Castaño A., Narotsky D.L., Hamid N., Khalique O.K., Morgenstern R., DeLuca A., Rubin J., Chiuzan C., Nazif T., Vahl T. (2017). Unveiling transthyretin cardiac amyloidosis and its predictors among elderly patients with severe aortic stenosis undergoing transcatheter aortic valve replacement. Eur. Heart J..

[36] Milani P., Dispenzieri A., Scott C.G., Gertz M.A., Perlini S., Mussinelli R., Lacy M.Q., Buadi F.K., Kumar S., Maurer M.S. (2018). Independent Prognostic Value of Stroke Volume Index in Patients with Immunoglobulin Light Chain Amyloidosis. Circ. Cardiovasc. Imaging.

[37] Siepen F.A.D., Bauer R., Voss A., Hein S., Aurich M., Riffel J., Mereles D., Röcken C., Buss S.J., Katus H.A. (2018). Predictors of survival stratification in patients with wild-type cardiac amyloidosis. Clin. Res. Cardiol..

[38] Barbieri A., Bursi F., Camaioni G., Maisano A., Imberti J.F., Albini A., De Mitri G., Mantovani F., Boriani G. (2021). Echocardiographic Left Ventricular Mass Assessment: Correlation between 2D-Derived Linear Dimensions and 3-Dimensional Automated, Machine Learning-Based Methods in Unselected Patients. J. Clin. Med..

